# Differential expression of Toll-like receptors on human alveolar macrophages and autologous peripheral monocytes

**DOI:** 10.1186/1465-9921-11-2

**Published:** 2010-01-05

**Authors:** Esmeralda Juarez, Carlos Nuñez, Eduardo Sada, Jerrold J Ellner, Stephan K Schwander, Martha Torres

**Affiliations:** 1 Departamento de Microbiología, Instituto Nacional de Enfermedades Respiratorias, (Calzada de Tlalpan) México City, (14080), México; 2 Servicio de Broncoscopia, Instituto Nacional de Enfermedades Respiratorias, (Calzada de Tlalpan) México City, (14080), México; 3 Center for Emerging & Reemerging Pathogens, University of Medicine and Dentistry New Jersey, (S Orange Ave), Newark, (07103), USA; 4 Department of Medicine, Section of Infectious Diseases, Boston Medical Center (Albany Street), Boston, (02118), USA; 5 Department of Environmental and Occupational Health, University of Medicine and Dentistry New Jersey - School of Public Health (Hoes Lane) Piscataway, (08854), USA; 6 Center for Global Public Health, University of Medicine and Dentistry New Jersey - School of Public Health (Hoes Lane) Piscataway, (08854), USA

## Abstract

**Background:**

Toll-like receptors (TLRs) are critical components in the regulation of pulmonary immune responses and the recognition of respiratory pathogens such as *Mycobacterium Tuberculosis (M.tb)*. Through examination of human alveolar macrophages this study attempts to better define the expression profiles of TLR2, TLR4 and TLR9 in the human lung compartment which are as yet still poorly defined.

**Methods:**

Sixteen healthy subjects underwent venipuncture, and eleven subjects underwent additional bronchoalveolar lavage to obtain peripheral blood mononuclear and bronchoalveolar cells, respectively. Surface and intracellular expression of TLRs was assessed by fluorescence-activated cell sorting and qRT-PCR. Cells were stimulated with TLR-specific ligands and cytokine production assessed by ELISA and cytokine bead array.

**Results:**

Surface expression of TLR2 was significantly lower on alveolar macrophages than on blood monocytes (1.2 ± 0.4% vs. 57 ± 11.1%, relative mean fluorescence intensity [rMFI]: 0.9 ± 0.1 vs. 3.2 ± 0.1, p < 0.05). The proportion of TLR4 and TLR9-expressing cells and the rMFIs of TLR4 were comparable between alveolar macrophages and monocytes. The surface expression of TLR9 however, was higher on alveolar macrophages than on monocytes (rMFI, 218.4 ± 187.3 vs. 4.4 ± 1.4, p < 0.05) while the intracellular expression of the receptor and the proportion of TLR9 positive cells were similar in both cell types. TLR2, TLR4 and TLR9 mRNA expression was lower in bronchoalveolar cells than in monocytes.

Pam3Cys, LPS, and *M.tb *DNA upregulated TLR2, TLR4 and TLR9 mRNA in both, bronchoalveolar cells and monocytes. Corresponding with the reduced surface and mRNA expression of TLR2, Pam3Cys induced lower production of TNF-α, IL-1β and IL-6 in bronchoalveolar cells than in monocytes. Despite comparable expression of TLR4 on both cell types, LPS induced higher levels of IL-10 in monocytes than in alveolar macrophages. *M.tb *DNA, the ligand for TLR9, induced similar levels of cytokines in both cell types.

**Conclusion:**

The TLR expression profile of autologous human alveolar macrophages and monocytes is not identical, therefore perhaps contributing to compartmentalized immune responses in the lungs and systemically. These dissimilarities may have important implications for the design and efficacy evaluation of vaccines with TLR-stimulating adjuvants that target the respiratory tract.

## Introduction

As a consequence of the physiological breathing process, lungs are the major portal of entry for airborne infectious microorganisms and environmental particulate matter. Pulmonary host defense mechanisms against these potential noxious insults rely in large part on coordinated local immune responses in the bronchoalveolar spaces of alveolar macrophages, lymphocytes, neutrophils, NK, NKT, γδ T cells and epithelial cells [1].

Alveolar macrophages are sentinel cells in the immune response against infectious pathogens in the lungs and involved in phagocytosis, antigen presentation, production of antimicrobial effector molecules, and release of cytokines and chemokines that in turn contribute to immune cell recruitment and activation [2-5]. The recognition of microorganisms by alveolar macrophages occurs through the sensory functions of pattern recognition receptors such as complement receptor 3 (CR3), c-type lectin Dectin-1, receptors for the Fc portion of IgG, scavenger receptors, chemokine receptors, mannose receptors, DC-SIGN, adenosine receptor and toll-like receptors (TLRs) [6-8].

Although TLRs are not implicated in the uptake of microorganisms, binding of their ligands activates monocytes, macrophages and dendritic cells, and triggers a host of innate and adaptive antimicrobial immune responses [4,9]. There are currently 11 known human TLRs [10,11], which are differentially expressed in distinct cell subsets and tissues. These TLRs recognize multiple components of microorganisms ranging from nucleic acids to complex proteins. Ligation of the TLRs triggers signaling pathways that involve the adaptor protein MyD88, activate the transcription factor NF-κB, and induce the release of proinflammatory cytokines or of secondary signals, which can be MyD88-independent [12-14]. TLR2, TLR4 and TLR9 are relevant in the recognition of mycobacterial antigens. For example in the mouse model of tuberculosis, 38 kDa glycolipid and PIM6 are sensed through TLR4 and have been found to trigger a protective type Th1 cytokine response in the lungs during *Mycobacterium tuberculosis (M.tb) *infection [15,16], whereas TLR2 ligation by mycobacterial liparabinomannan modulates inflammatory responses in mouse macrophages [17]. Moreover, potent immune response induced by mycobacterial DNA (*M.tb *DNA) through TLR9 has recently been described in mouse macrophages [18]. TLRs therefore play a critical role in the immune response against *M.tb*.

Tissue-specific TLR expression patterns are believed to reflect unique adaptations to the requirements within tissues for efficient innate immune responses under the special local exposure conditions to the external environment. Indeed, the expression of TLRs differs considerably between cell types and tissues in humans and mice [19,20]. For example, human peripheral blood monocytes and macrophages from lung tissue or colon express TLR1, TLR2, TLR3, TLR4 and TLR5 [20], whereas gut epithelial cells express TLR3 and TLR5 only [21].

TLR2 mRNA and surface expression has been described in human alveolar macrophages and lung epithelial cells from tumor-free lobectomy material of lung cancer patients [22]. TLR1, TLR2, and TLR4 expression was found on lymphocytes, myeloid cells and type II pneumocytes from granulomas of TB patients by immunocytochemistry, whereas TLR9 expression was restricted to macrophages and lymphocytes [23]. The same study found that TLR3 and TLR5 were expressed exclusively on alveolar macrophages and that TLR2 and IL-4 expression were inversely correlated. The latter suggests that TLR expression patterns may affect the profile of local host immune responses and Th immunity [23].

However, the expression of TLRs on human alveolar macrophages has remained ill-defined despite their presumed importance in protective immune responses against airborne pathogens such as *M.tb*. The present work therefore aimed at characterizing the expression of TLR2, TLR4 and TLR9 on human alveolar macrophages. Alveolar macrophages from healthy volunteers were compared with their autologous blood monocytes and monocyte-derived macrophages. A differential expression profile of the TLRs on the alveolar macrophages and monocytes emerged. Alveolar macrophages expressed lower levels of TLR2, comparable levels of TLR4, and higher levels of TLR9 than monocytes. These findings suggest that the capability of immune cells to recognize infectious pathogens or noxious particulate matter may be tissue and thus compartment-specific.

## Materials and methods

### Study subjects

Sixteen healthy persons (HIV-1 seronegative, with normal chest radiographs), three female, thirteen male, with a mean age of 29 ± 7 years, residents of Mexico City, were recruited by advertisement at the National Institute for Respiratory Diseases (INER) in Mexico City. Five of the study subjects were tuberculin skin test positive and 11 were tuberculin skin test negative. All study subjects underwent a venipuncture, and 11 of the 16 subjects underwent an additional fiberoptic bronchoscopy with bronchoalveolar lavage. Approval to perform these studies was given by the Institutional Review Boards of INER and the University of Medicine and Dentistry New Jersey (UMDNJ). Written informed consent was obtained prior to any procedures from all study subjects according to the guidelines of the U.S. Department of Health and Human Services.

### Culture medium

Unless otherwise specified, cells were cultured in RPMI 1640 (Cambrex, Walkersville, MD) supplemented with 50 μg/mL gentamycin sulfate, 200 mM L-glutamine and 10% heat-inactivated pooled human AB serum (Gemini Bioproducts, Sacramento, CA) at 37°C in 5% CO_2_.

### Preparation of bronchoalveolar cells

Bronchoalveolar cells were obtained by bronchoalveolar lavage as described previously [24]. Briefly, after local anesthesia of the upper airways with 2% lidocaine a flexible bronchoscope (P30, Olympus BF, New Hyde Park, NY) was introduced into the nose, throat and trachea with further instillation of 1% lidocaine to prevent coughing. The bronchoscope was wedged into the right middle lobe or the lingula and 150 mL of 0.9% sterile saline fluid instilled in 20-30 mL aliquots into each of two adjacent lung subsegments. Bronchoalveolar lavage fluid was centrifuged at 400 × g for 15 minutes at 4°C. Pellets of bronchoalveolar cells were resuspended in culture medium and viability of the bronchoalveolar cells assessed by Trypan blue exclusion (>98% in all cases). Bronchoalveolar cells were 95 ± 2.6% alveolar macrophages by flow cytometry using a gate based on size, granularity and HLA-DR expression. Basal TLR expression levels on alveolar macrophages were determined on freshly isolated bronchoalveolar cells within 2-4 hours of the bronchoalveolar lavage procedure.

### Preparation of peripheral blood mononuclear cells and purification of monocytes

Peripheral blood mononuclear cells were obtained from heparinized venous whole blood by gradient centrifugation over Ficoll (Axis-Shield PoC As, Oslo, Norway) using standard procedures [25]. Monocytes were obtained by positive selection from peripheral blood mononuclear cells using magnetic CD14^+ ^microbeads (Miltenyi Biotec, Auburn, CA) according to the manufacturer's instructions. Monocytes were washed twice and resuspended in culture medium. Viability of the monocytes was assessed by Trypan blue exclusion and was >98% in all cases. CD14 expression was greater than 90% (91.4% ± 1.9). Basal TLR expression was assessed by flow cytometry on these freshly isolated monocytes.

### Preparation of monocyte-derived macrophages

Monocytes were adjusted at 10^6 ^cells/mL in three mL culture medium and incubated in six-well plates for one, four and seven days. Cells were harvested using cell lifters (Corning Inc., Acton, MA), resuspended in culture medium, and used for flow cytometry and production of cell lysates for qRT-PCR.

### Culture and TLR staining of HEK293 cells

To assure specificity of binding of the TLR mABs, stably TLR-transfected human embryonic kidney cells (HEK293, kindly provided by Dr. Golenbock, University of Massachusetts) were used as positive controls. HEK293 cells were transfected with two types of fluorescent fusion proteins (YFP and CFP) fused to TLRs at the C-terminus: TLR2-YFP, TLR4-YFP and TLR9-CFP [26,27]. HEK293 cells were cultured in DMEM medium (Cambrex, Walkersville, MD) containing 4.5 g/L Glucose, 200 mM L-glutamine, 10% fetal bovine serum (Hyclone, Logan, Utah), 0.5 mg/mL G418-sulfate (MP Biomedicals, Solon, Ohio), 3.7 g/l sodium bicarbonate and 10 μg/mL Ciprofloxacin (Senosiain, Celaya, Mexico). HEK293 cells were harvested and stained for membrane and intracellular TLR detection with phycoerythrine (PE)-coupled anti-TLR2, TLR4 and TLR9 monoclonal and matched isotype control antibodies (all from eBioscience, San Diego, CA). Cells were subsequently fixed with 1% paraformaldehyde and kept at 4°C until acquisition of 20,000 cells with a FACSCalibur flow cytometer (Becton Dickinson, BD, San José, CA) within 24 hours. Flow cytometry was performed using a morphologic gate set on large granular cells (high FSC and SSC) with fluorescence detection in the PE (FL2) channel. This allowed discriminating fluorescence emitted from YFP and CFP-expressing TLR-transfected HEK cells.

TLR2 and TLR4-transfected HEK293 cells expressed TLR2 and TLR4 on their surfaces only. TLR9 transfected HEK293 cells expressed intracellular TLR9 only (as previously reported [27]). TLR2, TLR4 and TLR9-transfected HEK293 cells were antibody positive in 90%, 80% and 99.9%, respectively. None of the antibodies showed nonspecific crossreactive binding.

### Preparation of *M.tb *DNA

*M.tb *DNA was prepared as described previously by our group [28,29]. Briefly, 10^9 ^*M.tb *H37 Rv bacteria were digested with 2 mg/mL proteinase K in lysis buffer (50 mM TRIS-1 mM EDTA-0.5% Tween 20) at 56°C in a water bath overnight. Genomic bacterial DNA was extracted using a chloroform: isoamyl alcohol (49:1) mixture, precipitated with sodium acetate-ethanol (1:30) and then dissolved in pyrogen-free sterile water and stored at -20°C in aliquots. Human DNA was prepared in the same way from 5 × 10^6 ^peripheral blood mononuclear cells and used as a negative stimulation control. Concentration and purity of mycobacterial and human DNA were determined by spectrophotometry. Both DNA preparations were lipopolysaccharide (LPS) free as determined by Limulus Amebocyte Lysate Assay (PyrogentPlus, Cambrex, Walkersville, MD).

### Stimulation of monocytes and bronchoalveolar cells with TLR ligands

To assess ligand-induced TLR expression of monocytes and bronchoalveolar cells, 10^6 ^cells were cultured in a final volume of 1 mL in duplicate wells in ultra-low attachment polystyrene 24-well plates (Corning Inc.). Cells were stimulated with 1 ng/mL synthetic lipoprotein Palmitylated N-acyl-S-diacylglyceryl Cysteine (Pam3Cys) (EMC Microcollections, Tübingen, Germany), 100 ng/mL LPS from *Escherichia coli *(Sigma, St Louis, Missouri), *M.tb *DNA (5 μg/mL), and human DNA (5 μg/mL) as control DNA. In a pilot study, cells were stimulated for periods of 10 min, 30 min, 1 h, 4 h, 6 h, 18 h, 20 h and 24 h to define the optimal incubation periods for each TLR ligand. Optimal incubation periods were defined by the time points at which ligand-induced TLR expression either increased or decreased relative to basal values and remained constant thereafter. Following stimulation, one set of cultures from monocytes and bronchoalveolar cells was harvested and prepared for flow cytometry, and one set for mRNA extraction.

To assess TLR ligand-induced cytokine production, 10^6 ^purified monocytes or bronchoalveolar cells were stimulated for 24 h in 24-well plates (Corning Inc) at the following final concentrations per mL: 1 ng Pam3Cys, 100 ng of LPS, 5 μg of mycobacterial DNA (*M.tb *DNA), and 5 μg of human DNA (control DNA). Culture medium alone was used as a negative control. TNF-α and IL-6 concentrations were determined in culture supernatants using in-house ELISAs [30]. Mouse anti-human TNF-α [1 μg/mL, Pharmingen, San Diego, CA], and anti-human IL-6 [2 μg/mL, R&D, Minneapolis, MN] were used as capture antibodies, mouse anti-human biotinylated anti-TNF-α0.5 μg/mL, Pharmingen], and anti-IL-6 [0.3 mg/mL, R&D]) as secondary detection antibodies. Standard curves (0-2000 pg/mL) were prepared with recombinant human cytokines (TNF-α, Endogen, Woburn, MA; IL6, R&D). IL-1β, IL-10 and IL-12 were assessed in 24-hour culture supernatants using the human inflammation cytokine bead array kit (BD Biosciences, San Jose, CA).

### Surface and Intracellular TLR Expression by Fluorescence Activated Cell Sorting

Surface expression levels of TLR2, TLR4 and TLR9 on human alveolar macrophages, autologous monocytes and monocyte-derived macrophages were determined by FACS analysis. Prior to specific antibody staining and in order to block nonspecific Fc receptor binding, 10^6 ^bronchoalveolar cells and monocyte-derived macrophages were incubated in 1 × phosphate buffered saline (Cambrex, Walkersville, MD) with 50% rabbit serum for 10 min at room temperature in agitation (30 rpm). Saturating amounts of phycoerythrin (PE)-labeled mAbs against TLR2, TLR4, TLR9 (eBioscience, San Diego, CA), HLA-DR and matching isotype control antibodies (BD PharMingen, San Diego, CA), were then added and incubated for 30 minutes at room temperature in the dark. Cells were then washed once with 1 × phosphate buffered saline by centrifugation at 600 × g for 5 minutes. Cells were subsequently fixed with 1% paraformaldehyde and kept at 4°C until acquisition of 20,000 cells with a FACSCalibur flow cytometer (Becton Dickinson, BD, San José, CA) within 24 hours. Flow cytometric analysis was performed using a morphologic gate set on large granular cells (high FSC and SSC). To assess the intracellular and cell surface expression of TLR9, cells were permeabilized (permeabilizing buffer, Becton Dickinson) or remained unpermeabilized, respectively. Macrophage autofluorescence was compensated by setting the PE detector voltage to a minimum level that discriminates between autofluorescence and specific staining in both negative and positive controls. Isotype control antibodies were used to define settings in histogram plot analyses. TLR expression of the cells is presented in two ways: as proportions of positive cells and as relative mean fluorescence intensity (rMFI) of the specific monoclonal antibody/mean fluorescence intensity of the corresponding isotype control.

### Reverse transcription and real-time PCR for TLR2, TLR4 and TLR9 gene expression

Total RNA was isolated from cell lysates of 10^6 ^unstimulated or of 10^6 ^ligand-stimulated monocytes and bronchoalveolar cells using RNAeasy Kit (Qiagen, Germantown, MD) according to manufacturer's protocol. DNAse-treated RNA was reverse transcribed using 2 μg of RNA and random hexamers following a protocol of the Superscript First-Strand Synthesis kit (Invitrogen, Carlsbad, CA) and subjected to quantitative PCR.

Quantitative real-time PCR (qRT-PCR, TaqMan) was performed to determine the relative TLR2, TLR4 and TLR9 mRNA expression levels using the comparative threshold cycle (ΔΔCt) method of relative quantitation (PerkinElmer User Bulletin no. 2). All real time PCR reagents were purchased from Applied Biosystems (Carlsbad, CA). Real time PCR reactions were performed in duplicate wells using 12.5 μl PCR master mix, 5 μl of cDNA and 1.25 μl of Taqman pre-designed gene assay for TLR2 (Hs00610101_m1), TLR4 (Hs00152939_m1) and TLR9 (Hs00152973_m1). Volumes were adjusted to 25 μl per well with RNAse free water. PCR cycles were as follows: 50°C for 2 min, 95°C for 10 min, followed by 40 cycles of 95°C for 15 s and 60°C for 1 min, on an ABI Prism 7500 Sequence Detection System (Applied Biosystems). Threshold values were set on the amplification plots, and the calculated Ct values were exported to Microsoft Excel for analysis. The Ct values for each gene were normalized to the endogenous control gene 18 S rRNA (4319413 E). The effect of DNA concentration on PCR efficiency was validated (PerkinElmer User Bulletin no. 2). To analyze the constitutive expression of each of the TLR genes in bronchoalveolar cells and monocytes, TLR gene expression in autologous monocytes was set as 1, and the TLR gene expression of the autologous bronchoalveolar cells reported relative to that of the monocytes. To analyze the ligand-induced TLR mRNA expression at 1 h and 24 h post-stimulation TLR mRNA expression of unstimulated bronchoalveolar cells and monocytes was set as 1, and the TLR mRNA expression of the ligand-stimulated cells reported relative to that of the unstimulated cells.

### Statistical analysis

Data were analyzed using the non-parametric two-tailed Wilcoxon signed-rank test. Means and standard errors (SEs) are presented. Statistical significance was set at p < 0.05. Analyses were done using SPSS 13.0 for Windows (SPSS, Chicago, IL, 2005).

## Results

### Alveolar macrophages express lower cell surface TLR2 and higher TLR9 levels than autologous monocytes

**Figure 1:**
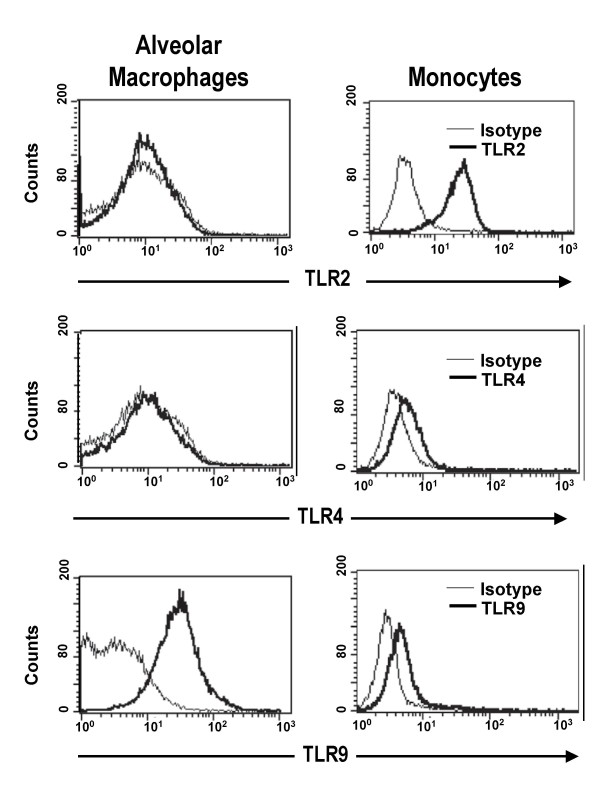
**Differential constitutive surface expression of TLR2, TLR4 and TLR9 on human alveolar macrophages and monocytes**. Alveolar macrophages and monocytes from healthy donors were analyzed by flow cytometry using phycoerythrin (PE)-coupled mouse anti-TLR2, TLR4 and TLR9 antibodies and their corresponding isotype controls (gray thin lines). Histograms are representative of eight independent experiments.

The proportion of TLR2-expressing cells and the rMFI levels of TLR2 by flow cytometry were significantly lower in alveolar macrophages than in monocytes (1.2 ± 0.4% vs. 57 ± 11.1% and 0.9 ± 0.1 vs. 3.2 ± 0.1, respectively, p < 0.05). The proportion of TLR4-expressing cells and rMFIs of TLR4 were comparable between alveolar macrophages and monocytes (1.3 ± 0.2% and 3 ± 0.8% and 1.1 ± 0.1 vs. 1.5 ± 0.2, respectively). To determine cell surface expression of TLR9, unpermeabilized alveolar macrophages and monocytes were assessed by flow cytometry. Interestingly, the proportion of alveolar macrophages that expressed TLR9 on their surface was similar to that of monocytes (54.6 ± 15.5% vs. 39.8 ±14.7%), however, the TLR9 rMFI, was significantly higher in alveolar macrophages than in monocytes (rMFI, 218.4 ± 187.3 vs. 4.4 ± 1.4, p < 0.05) (Figure 1 and Table 1). The expression of intracellular TLR9 was comparable in both monocytes and alveolar macrophages (data not shown).

**Table 1:** Constitutive surface expression of TLR2, TLR4 and TLR9

Receptor	% Cells expressing TLRs			Cell Surface Expression (rMFI)		
	**MN**	**AM**	**MDM**	**MN**	**AM**	**MDM**

**TLR2**	57 ± 11.1	1.2 ± 0.4*	1.5 ± 1.2*	3.2 ± 0.1	0.9 ± 0.1*	1.4 ± 0.5*

**TLR4**	3.0 ± 0.8	1.3 ± 0.2	3.8 ± 1.4	1.5 ± 0.2	1.1 ± 0.1	1.3 ± 0.3

**TLR9**	39.8 ± 14.7	54.6 ± 15.5	38 ± 20	4.4 ± 1.4	218.4 ± 187.3*	3.6 ± 0.9

**Constitutive surface expression of TLR2, TLR4 and TLR9 on human monocytes and alveolar macrophages and monocyte-derived macrophages**. TLR levels were determined on monocytes (MN, n = 8), alveolar macrophages (AM, n = 7) and monocyte-derived macrophages (MDM, n = 8) by flow cytometry. Results present mean percentages ± SE of cells expressing TLR and relative mean fluorescence index (rMFI) ± SE as a measure of the TLR expression density. (*) statistically significant differences compared to monocytes (p < 0.05).

### TLR2 expression is modified during the monocyte differentiation process

**Figure 2:**
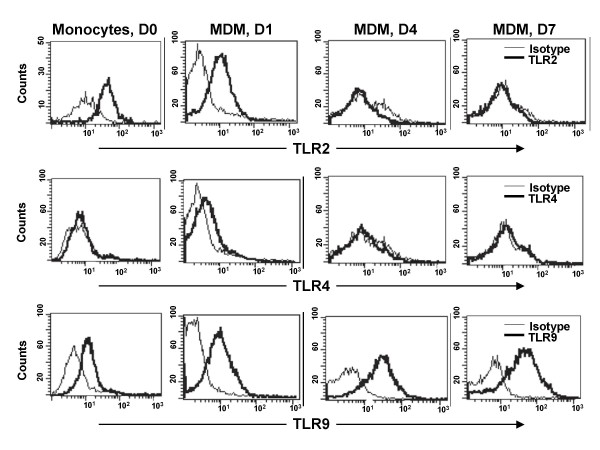
**Modulation of TLR2, TLR4, and TLR9 expression during macrophage maturation**. Monocyte-derived macrophages (MDM) were obtained from monocytes during a 7-day culture period in plastic dishes. Surface TLR expression was assessed by flow cytometry on freshly isolated monocytes (D0) and on cultured monocytes after 1 day (D1), 4 days (D4) and 7 days (D7) of differentiation. Histograms are representative of five independent experiments.

The observed differences in TLR expression levels between alveolar macrophages and monocytes may have resulted from differences in the source tissue microenvironment or the maturation stages of the cells. To test the latter possibility, we modeled the impact of the differentiation process from monocytes to macrophages on the expression of TLRs by *in vitro *monocyte maturation. Expression levels of TLR2, TLR4 and TLR9 were monitored by flow cytometry in the transition process from monocytes to monocyte-derived macrophages. Interestingly, TLR2 surface expression (rMFI) and the proportion of TLR2 positive cells decreased after 24 hours of culture in Petri dishes and through day 7 (D7) when cells portrayed a macrophage phenotype as determined by light microscopy (Day 0, basal rMFI 3.9 ± 0.9, 54 ± 10.4%; Day 4 rMFI 1.4 ± 0.36, 8.5 ± 7.8%, Day 7 rMFI 1.4 ± 0.5, 1.5 ± 1.2%, p < 0.05). TLR4 expression remained unchanged during the differentiation of monocytes into macrophages (D0, rMFI 1.3 ± 0.2, D4 rMFI 1.25 ± 0.22, D7 rMFI 1.3 ± 0.3) while the expression of TLR9 varied although not statistically significant (D0, basal rMFI 6.3 ± 1.2, rMFI at D1, 3 ± 0.6, rMFI at D4 4.85 ± 1.43, rMFI at D7 3.6 ± 0.9) (Figure 2 and Table 1).

### TLR2, TLR4 and TLR9 mRNA expression in monocyte-derived and alveolar macrophages

**Figure 3:**
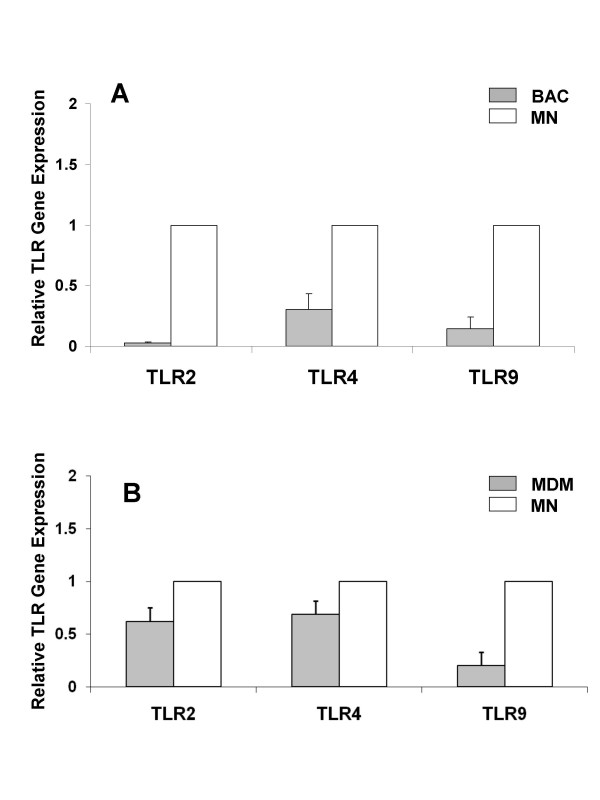
**Bronchoalveolar cell mRNA expression of TLR2, TLR4 and TLR9 is lower than that of monocytes**. TLR gene expression in unstimulated cells was assessed by qRT-PCR and relative quantification determined using the ΔΔCT method. Gene expression was normalized to 18 S rRNA. TLR expression of bronchoalveolar cells (BAC, panel A) and monocyte-derived macrophages (MDM, panel B) is reported relative to monocytes (MN). TLR expression on monocytes was set at 1. Depicted are mean ± SE of five individuals.

The mRNA expression levels of TLRs were assessed by qRT-PCR (TaqMan) in alveolar macrophages and monocytes using the ΔΔCt method allowing a comparison of the TLR mRNA expression of alveolar macrophages relative to that of monocytes. The expression of TLR2, TLR4 and TLR9 mRNA of alveolar macrophages was lower than that of autologous monocytes (Figure 3A).

To determine the TLR mRNA expression during monocyte differentiation into macrophages, mRNA from monocyte cultures during seven-day plastic adherence was extracted and TLR mRNA expression of monocyte-derived macrophages was assessed relative to that of autologous monocytes on day 0. Monocyte-derived macrophages expressed lower TLR2, TLR4 and TLR9 mRNA levels than monocytes thus resembling alveolar macrophages (Figure 3B).

### Regulation of TLR surface expression in response to TLR ligands in monocytes and alveolar macrophages

**Figure 4:**
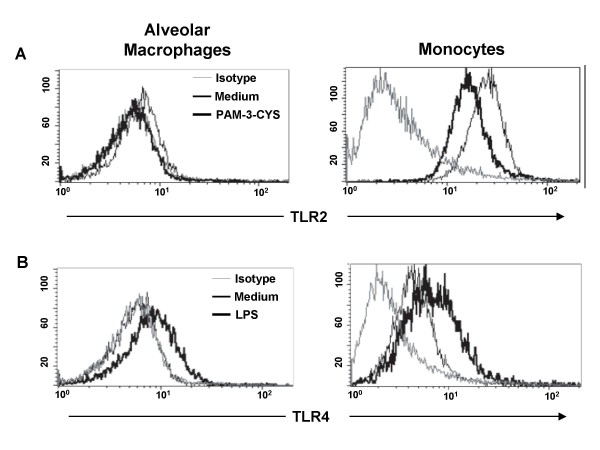
**TLR expression upon ligand recognition**. Alveolar macrophages and monocytes were cultured for 30 minutes in presence of 1 ng/mL Pam3Cys (TLR2, panel A). Alveolar macrophages and monocytes were cultured for 10 minutes in presence of 100 ng/mL LPS (TLR4, panel B). TLR expression after ligand stimulation was determined by flow cytometry. Histograms are representative of six independent experiments.

To assess the expression of TLR2, TLR4 and TLR9 by flow cytometry following ligand exposure, alveolar macrophages and monocytes were stimulated for the optimal incubation periods (described in the Methods section) with Pam3Cys (30 minutes), LPS (10 minutes) and *M.tb *DNA (24 hours), respectively. Following the 30-minute-exposure to Pam3Cys, TLR2 expression levels on alveolar macrophages remained unchanged, whereas on monocytes it was decreased below constitutive (culture medium) levels in all the individuals tested (Figure 4A).

Stimulation of alveolar macrophages and monocytes with LPS, however, augmented the expression of TLR4 on both alveolar macrophages and monocytes already after 10 minutes (Figure 4B). No further changes of TLR2 and TLR4 surface expression had been observed within a 24-hour observation period in our pilot study (data not shown). TLR9 expression after *M.tb *DNA stimulation was reduced in monocytes from six of nine and in alveolar macrophages from seven of nine subjects after 24 hours, however, statistical significance was not reached (data not shown). Cell exposure to human DNA did not alter the expression of TLR9 (data not shown).

### Regulation of TLR mRNA expression by TLR specific ligands

To determine whether cellular activation may regulate TLR mRNA levels, cells were stimulated with LPS, Pam3Cys and *M.tb *DNA, for 1 h and 24 h, respectively. Total RNA was extracted from the cells and analyzed by qRT-PCR.

**Figure 5:**
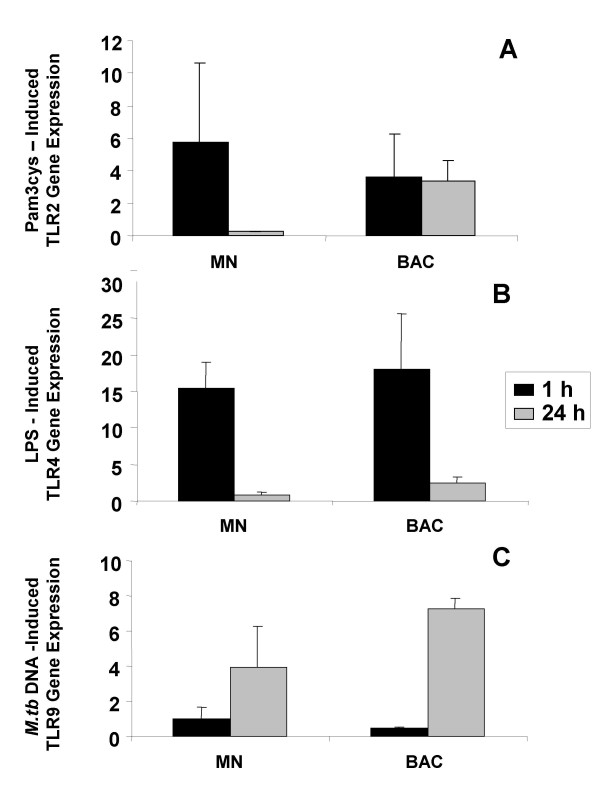
**Regulation of TLR mRNA expression after ligand exposure**. Bronchoalveolar cells (BAC) and monocytes (MN) were incubated in presence of 1 ng/ml of Pam3Cys and 100 ng/ml of LPS during 1 h or 24 h. Total RNA from cell lysates was reverse transcribed and qRT-PCR performed to quantify mRNA expression. Ligand-induced TLR2, TLR4 and TLR9 expression is reported relative to that of the unstimulated autologous cells. Mean ± SE of five independent experiments are depicted.

Pam3Cys upregulated the expression of TLR2 mRNA in monocytes within a 1-hour incubation period only, whereas in alveolar macrophages TLR2 mRNA upregulation was detected after 1 h and then maintained until 24 h (Figure 5A).

LPS upregulated TLR4 mRNA in both monocytes and alveolar macrophages after 1 h only, and was decreased below basal levels in both cells types after 24 h (Figure 5B).

*M.tb *DNA, in contrast, upregulated TLR9 mRNA in monocytes and alveolar macrophages after 24 h only (Figure 5C). These observations suggest that the expression of TLR2, TLR4 and TLR9 may be regulated differentially *in vivo *at sites of infection or inflammation by bacterial components or TLR specific ligands. There were no differences noted in the cell surface expression or the mRNA levels of TLR2, TLR4, and TLR9 or the responsiveness of the TLRs to their ligands between cells from TST positive (n = 4) and TST negative (n = 7) subjects.

### TLR ligands induce production of pro-inflammatory cytokines by bronchoalveolar cells and monocytes

**Figure 6:**
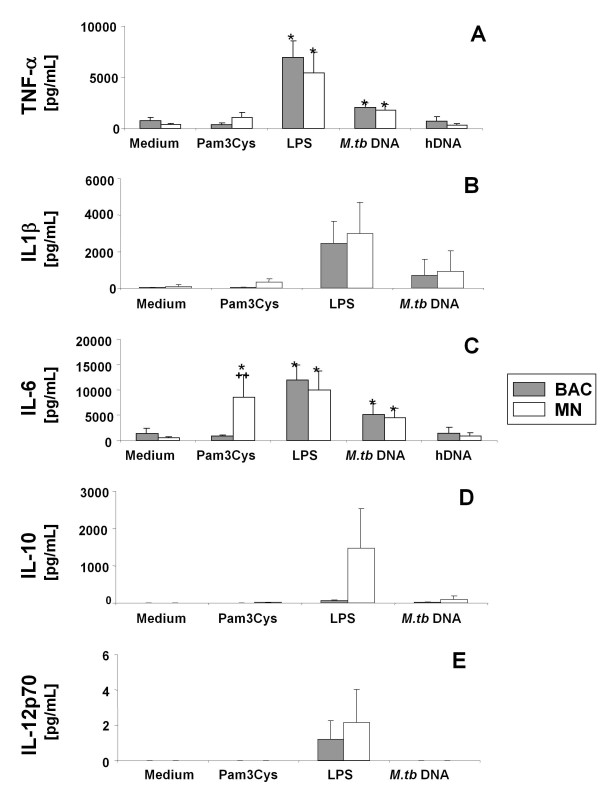
**TLR-ligands induce differential cytokine production**. Bronchoalveolar cells (BAC) and monocytes (MN) were stimulated with 5 mg/mL DNA from *M.tb *H37 Rv, 100 ng/mL LPS and 1 ng/mL Pam3Cys for 20 hours. Cytokines were determined in culture supernatants by ELISA for TNF-α and IL-6 (**A, C**) and Cytokine Bead Array for IL-1β IL-10 and IL-12 (**B, D **and **E**). Culture medium and human DNA (5 mg/mL) were used as negative stimulation controls. Mean cytokine pg/mL ± SE of seven independent experiments are depicted. Statistically significant differences (p < 0.05): (*) comparing stimulated vs. controls (medium) within respective cell groups, (++) comparing bronchoalveolar cells with monocytes.

To assess the cytokine-inducing functional capability of TLR2, TLR4 and TLR9, we assessed the release of TNF-α, IL-1β, IL-6, IL-10 and IL-12 following ligand-stimulation of bronchoalveolar cells (95 ± 2.6% alveolar macrophages) and monocytes in response to Pam3Cys, LPS, *M.tb*-DNA, and human DNA and culture medium (control). Stimulation with Pam3Cys showed a trend towards lower TNF-α production levels (mean ± SD [pg/mL], 376 ± 152 versus 1080 ± 495, Figure 6A) and significantly lower levels of IL-6 (887 ± 150 versus 8485 ± 4548, p < 0.05, Figure 6C) in bronchoalveolar cells than in monocytes. Levels of IL-1β (Figure 6B) were comparably low (mean ± SD [pg/mL], IL-1β: 27.8 ± 18.1 versus 333.8 ± 179.0) and levels of IL-10 and IL-12 undetectable (Figure 6D and 6E). These findings coincided with the lower surface expression levels of TLR2 on bronchoalveolar cells compared with monocytes and suggested that Pam3Cys may preferentially induce the production of TNF-α and IL-6.

LPS induced similar levels of TNF-α, IL-1β IL-6 and IL-12 in bronchoalveolar cells and monocytes, (mean ± SD [pg/mL], TNF-α 6915 ± 1675 versus 5436 ± 2008, IL-1β 3653.8 ± 1695.6 versus 2459.1 ±1211, IL-6: 11931 ± 2983 versus 9985 ± 3770, IL-12: 1.7 ± 0.7 versus 2.8 ± 1.2, Figure 6A, B and 6E, respectively) while the induction of IL-10 was significantly lower in bronchoalveolar cells than in monocytes (mean ± SD [pg/mL], IL-10: 65.4 ± 14.6 versus 1471.6 ± 250.8, p < 0.05).

*M.tb*-DNA induced comparable levels of TNF-α, IL-1β and IL-6 but did not induce IL-10 or IL-12 in bronchoalveolar cells and monocytes (mean ± SD [pg/mL], TNF-α: 2049 ± 421 and 1779 ± 560; IL-1β: 910.3 ± 1138.9 and 700.3 ± 899; IL-6: 5142 ± 2153 and 4485 ± 1922, respectively, Figure 6A, B, C). Culture medium alone or human DNA (control stimuli) induced comparably low levels of all the cytokines (<30 pg/mL) studied in both cell types.

## Discussion

The expression profile of TLRs and its potential contribution to human innate pulmonary immune responses in the alveolar spaces in response to bacterial components are poorly understood. We therefore compared the constitutive and ligand-induced expression of TLR2, TLR4 and TLR9 that are involved in the recognition of *M.tb *on alveolar macrophages, with that on autologous blood monocytes and monocyte-derived macrophages from healthy persons.

Resting human alveolar macrophages were characterized by significantly lower TLR2 and comparably low TLR4 surface expression levels than autologous monocytes. The flow cytometry findings for TLR2 were consistent with the mRNA expression levels in the current work. These findings also coincide with reports of five-fold decreased TLR2 mRNA levels in healthy lung tissues compared to that in human peripheral leukocytes [20,31], and with lower TLR2 mRNA levels in human alveolar macrophages than in autologous monocytes [32]. Our observation of reduced TLR2 surface expression on alveolar macrophages coincides functionally with the lower production of TNF-α and IL-6 following Pam3Cys stimulation of bronchoalveolar cells compared with autologous monocytes.

TLR4 cell surface expression was low and comparable in alveolar macrophages and monocytes, and TLR4 mRNA lower in alveolar macrophages than monocytes. These discrepancies may be explained by differences in the time kinetics of TLR4 trafficking and surface expression and mRNA expression. Nevertheless, despite the low expression levels of TLR4 in alveolar macrophages, these cells produced significantly higher (p < 0.05) amounts of IL-1β, IL-6 and TNF-α in response to LPS than to culture medium. This suggests that small expression levels of TLR4 may suffice to induce cytokine production and TLR4 mRNA expression. Intriguingly, TLR9 surface expression detected by flow cytometry was 50-fold greater on resting primary alveolar macrophages than on primary autologous monocytes, although the proportion of cells expressing the receptor and the intracellular expression levels were similar. This observation contrasts the notion that TLR9 is expressed primarily intracellular, as was previously suggested by some authors in macrophages and dendritic cells [33-35]. The findings in the current study and that of other authors [36-38], however, provide evidence that the expression of TLR9 may in fact be both, intracellular and on the cell surface. The higher expression density of TLR9 on the cell surface of the alveolar macrophages (compared with that on the monocytes) was inconsistent with the lower TLR9 mRNA expression of these cells. This may for example be due to the half life of the receptors, or dissociation between TLR9 trafficking and de novo protein synthesis in the two cell types.

Because the distinct expression levels of TLR2 found on alveolar macrophages and monocytes may have been due to differences in the maturation stages of these cells we assessed monocyte-derived macrophages in parallel. We had previously reported that monocyte-derived macrophages obtained under plastic adherence culture conditions resemble alveolar macrophages in their capacity to phagocytose *M.tb *and to express LL-37 [29]. In the current study, we found by flow cytometry and qRT-PCR that TLR2 was downregulated within 24 hours of monocyte culture and remained low throughout the seven-day differentiation period into macrophages. Interestingly, the low TLR2 expression levels on monocyte-derived macrophages on day seven coincided with the low constitutive TLR2 expression found on alveolar macrophages (Figures 1 and 2). These results are also compatible with those from Henning et al who reported a significant reduction of TLR2 protein and mRNA, and unaltered TLR4 expression during the *in vitro *maturation of human monocytes to macrophages in Teflon wells [39]. Thus, the low-level expression of TLR2 appears to be a feature of primary alveolar macrophages as well as of *in vitro *generated monocyte-derived macrophages. In contrast, induction of macrophage maturation by M-CSF, has been shown to result in unchanged TLR2, increased TLR4 and very low TLR9 mRNA expression levels [40]. Macrophage TLR expression assessed in experimental culture microenvironments thus depends on the presence or absence of a variety of factors, including type and concentrations of cytokines and of additional proteins such as surfactant protein A [39].

We also assessed the effects of TLR-specific ligands on the expression of TLR2, TLR4 and TLR9, as both, TLR ligands and cytokines have been reported to regulate TLR expression [31,41].

TLR2 cell surface expression by flow cytometry was decreased on monocytes after stimulation with Pam3Cys, whereas the expression of TLR2 on alveolar macrophages in response to Pam3Cys remained unchanged. Pam3Cys induced TLR2 mRNA expression was increased as early as after 1 h in both cells types, but was maintained for a longer time in bronchoalveolar cells. These findings suggest that TLR2 may be differentially regulated in monocytes and alveolar macrophages.

TLR4, in contrast was shown to be upregulated in response to LPS on monocytes and alveolar macrophages in a kinetic similar to that of TLR2 using both flow cytometry and qRT-PCR. Taken together these results indicate a differential, cell-type-specific ligand-mediated regulation of the expression of TLR2 and TLR4.

It was beyond the scope of this study to assess in detail whether ligand-binding alone, and/or cytokine release in the cellular microenvironment affected the regulation of the TLRs. While TLR2 regulation may be due to Pam3Cys ligation and/or cytokine production from macrophages or other cellular subsets within the bronchoalveolar cells (5-8% are lymphocytes), regulation of TLR4 expression may result from a direct effect of LPS on the cell membrane as it was noted already within 10 minutes of LPS stimulation.

TLR9 cell surface expression detected by flow cytometry in response to *M.tb *DNA did not show a uniform pattern, however, was diminished on alveolar macrophages and on monocytes in 65% to 75% of all study subjects. We speculate that this phenomenon may be due to the internalization of cell surface TLR9 after binding to its ligand. Alternatively, TLR9 may become undetectable to the antibodies used during the flow cytometry after binding to its ligand. TLR9 mRNA was upregulated after 24 hours of incubation with *M.tb *DNA in both monocytes and alveolar macrophages in five of five study subjects indicating a slower kinetic of TLR9 mRNA generation than in the case of TLR2 and TLR4.

We also assessed the induction of cytokines following stimulation of the cells with TLR-specific ligands. TNF-α and IL-6-production levels after Pam3Cys (TLR2) stimulation were significantly lower in bronchoalveolar cells than in autologous monocytes. This data is consistent with the lower TLR2 expression levels found on alveolar macrophages in the current study and with data from a recent study in which lipoteichoic acid, a TLR2 ligand, was instilled experimentally into lung segments of human volunteers and resulted in a poor transcription of IL-1β, IL-6, and IL-8 genes [42].

In the current study, TNF-α, IL-1β and IL-6 production in response to LPS (TLR4) was not significantly different between bronchoalveolar cells and monocytes. However, LPS induced higher production of IL-10 in monocytes than in bronchoalveolar cells suggesting that activation via TLR4 may result in differential cytokine production from alveolar macrophages and monocytes. This observation may find a mechanistic explanation in a study of human alveolar macrophages in which LPS-induced IL-10 production was associated with a reduced capacity to activate STAT3. This suggested that TLR ligand activation may modulate the anti-inflammatory activity of IL-10 either by altering its production or by inhibiting the cellular responsiveness to the cytokine [43].

*M.tb *DNA (TLR9) induced similar levels of TNF-α, IL-1β and IL-6 in bronchoalveolar cells and monocytes although the density of TLR9 expression was higher on alveolar macrophages than monocytes. This finding may suggest expression of non-functional cell surface TLR9, while the comparable induction of cytokine production in both cell types suggests that intracellular TLR9 may be the functional component of the receptor. One may speculate that the cell surface form of TLR9 binds bacterial DNA (released from infected and dying cells) and that the ligand is then transferred from the cell surface into the intracellular compartment. This may provide an extra safety step prior to inducing the inflammatory cascade. Indeed, there is evidence that for TLR9 to be functional, an ectodomain cleavage in the endolysosome is required to recruit MyD88 upon activation, and that a truncated rather than the full-length form of the receptor is functional. Both, full-length and cleaved forms of TLR9, however, are capable of binding ligand [44].

Tissue compartment-specific immune responses thus may be characterized by differential expression levels of TLRs, such as those shown here for TLR2, that result in distinct production levels of cytokines. These immunoregulatory mechanisms may shape the inflammatory cytokine response and thus the potential of damage to the tissue that, as in the case of the lungs, is exposed continuously to inhaled pathogens and noninfectious particulate matter. A suppressive immunoregulatory mechanism involving TLRs has been described recently as alveolar surfactant protein A (SP-A) was shown to downregulate TLR2 expression and TNF-α production in human macrophages [39].

Limitations of the current study are related to the difficulty to recruit healthy volunteers for lung immunity studies and the resulting small study subject numbers. *In vitro *studies also may not reflect exactly processes *in vivo*. Further, the bronchoalveolar cells contained a small proportion (5-8%) of alveolar lymphocytes of which a small subset may express TLRs [45-47]. Similarly, monocyte populations contained up to 10% of contaminating lymphocytes, despite plastic adherence and magnetic bead enrichment. It is thus possible that a small component of TLR ligand-induced cytokines derived from lymphocytes and not from alveolar macrophages or monocytes.

## Conclusions

The observations in this study have clinical implications. Differences in the expression profile of TLRs between the blood and lung compartments may have to be considered for the design and efficacy evaluation of new vaccine-adjuvant combinations. New vaccines against respiratory pathogens, such as *M.tb*, may target the respiratory system to provide optimal protection locally in the near future.

## Competing interests

The authors declare that they have no competing interests.

## Authors' contributions

EJ carried out the cell culture flow cytometry assays and prepared the first draft of the manuscript.

CN carried out the bronchoalveolar lavages.

ES participated in the preparation of the manuscript

JJE participated in the preparation of the manuscript and was the PI of the NIH grant that supported much of this project.

SKS co-developed the study idea, participated in the design of the study and the experimental work, and spearheaded the final preparation of the manuscript.

MT co-developed the study idea, participated in the design of the study and the preparation of the manuscript and coordinated the experimental work.

All authors have read and approved the final manuscript.
